# Daily Step Count Predicts Acute Exacerbations in a US Cohort with COPD

**DOI:** 10.1371/journal.pone.0060400

**Published:** 2013-04-04

**Authors:** Marilyn L. Moy, Merilee Teylan, Nicole A. Weston, David R. Gagnon, Eric Garshick

**Affiliations:** 1 Department of Veteran Affairs, Veterans Health Administration, Rehabilitation Research and Development Service, Washington, D. C., United States of America; 2 Pulmonary and Critical Care Medicine Section, VA Boston Healthcare System, Boston, Massachusetts, United States of America; 3 Division of Pulmonary and Critical Care Medicine, Department of Medicine, Brigham and Women's Hospital, Boston, Massachusetts, United States of America; 4 Channing Division of Network Medicine, Department of Medicine, Brigham and Women's Hospital, Boston, Massachusetts, United States of America; 5 Harvard Medical School, Boston, Massachusetts, United States of America; 6 Department of Biostatistics, Boston University School of Public Health, Boston, Massachusetts, United States of America; 7 VA Cooperative Studies, Boston, Massachusetts, United States of America; Pulmonary Research Institute at LungClinic Grosshansdorf, United States of America

## Abstract

**Background:**

COPD is characterized by variability in exercise capacity and physical activity (PA), and acute exacerbations (AEs). Little is known about the relationship between daily step count, a direct measure of PA, and the risk of AEs, including hospitalizations.

**Methods:**

In an observational cohort study of 169 persons with COPD, we directly assessed PA with the StepWatch Activity Monitor, an ankle-worn accelerometer that measures daily step count. We also assessed exercise capacity with the 6-minute walk test (6MWT) and patient-reported PA with the St. George's Respiratory Questionnaire Activity Score (SGRQ-AS). AEs and COPD-related hospitalizations were assessed and validated prospectively over a median of 16 months.

**Results:**

Mean daily step count was 5804±3141 steps. Over 209 person-years of observation, there were 263 AEs (incidence rate 1.3±1.6 per person-year) and 116 COPD-related hospitalizations (incidence rate 0.56±1.09 per person-year). Adjusting for FEV_1_ % predicted and prednisone use for AE in previous year, for each 1000 fewer steps per day walked at baseline, there was an increased rate of AEs (rate ratio 1.07; 95%CI = 1.003–1.15) and COPD-related hospitalizations (rate ratio 1.24; 95%CI = 1.08–1.42). There was a significant linear trend of decreasing daily step count by quartiles and increasing rate ratios for AEs (*P* = 0.008) and COPD-related hospitalizations (*P* = 0.003). Each 30-meter decrease in 6MWT distance was associated with an increased rate ratio of 1.07 (95%CI = 1.01–1.14) for AEs and 1.18 (95%CI = 1.07–1.30) for COPD-related hospitalizations. Worsening of SGRQ-AS by 4 points was associated with an increased rate ratio of 1.05 (95%CI = 1.01–1.09) for AEs and 1.10 (95%CI = 1.02–1.17) for COPD-related hospitalizations.

**Conclusions:**

Lower daily step count, lower 6MWT distance, and worse SGRQ-AS predict future AEs and COPD–related hospitalizations, independent of pulmonary function and previous AE history. These results support the importance of assessing PA in patients with COPD, and provide the rationale to promote PA as part of exacerbation-prevention strategies.

## Introduction

COPD is the fourth most common cause of death in the US, affects 5% of US adults, and accounts for a large number of hospitalizations [1], [2]. Persons with COPD have significantly reduced exercise capacity, measured by clinic-based tests such as the 6-minute walk test (6MWT), and reduced physical activity (PA), directly measured with accelerometers or assessed by questionnaires [3]–[10]. In addition, lower 6MWT distance and lower daily step count, a direct and novel measure of PA, are significant predictors of all-cause mortality in COPD [11], [12].

COPD is also characterized by acute exacerbations (AEs) which result in poorer health-related quality of life (HRQL), a faster decline in lung function, and increased mortality [13]–[17]. Hospitalizations due to AEs account for a large portion of COPD-related medical costs [18]. To date, the key known factors predicting AEs and hospitalizations are the degree of airflow obstruction and history of prior AEs [13], [19]–[23]. Since these factors are usually already maximized with medical pharmacological therapy, there is a need to identify additional factors that can be targeted for intervention [20].

Persons with COPD have a wide range of PA levels which may be potentially modifiable. The relationship between PA and risk of AEs and COPD-related hospitalizations is unclear [24]. Prior studies have been limited because they assessed PA only by self-report which is notoriously overestimated, did not account for prior AE history, or studied only severe AEs that resulted in hospitalizations [8], [9], [19], [25]–[26]. In this report, our primary aim is to examine the relationship between PA directly measured with an accelerometer and risk of moderate and severe AEs and COPD-related hospitalizations. We hypothesize that lower daily step count predicts greater risk of AEs and COPD-related hospitalizations, independent of lung function and prior AEs. As a secondary aim, we assess 6MWT, a commonly used clinic-based test of exercise capacity, and patient-report of PA with the St. George's Respiratory Questionnaire Activity Score (SGRQ-AS), and examine their relationships with risk of AEs and COPD-related hospitalizations.

## Methods

### Ethics Statement

The protocol was approved by the VA Boston Healthcare System Committee on Human Research, and written informed consent obtained from each participant.

### Study Design and Participants

This work arises from a study which has been previously published, and the subjects reported here include the 127 subjects previously reported [27]. Between January 2009 and November 2011, we recruited eligible participants who were over 40 years of age and who had received care for COPD in the VA Boston Healthcare System general pulmonary clinics. The diagnosis of COPD was defined as having a smoking history of at least 10 pack-years and a ratio of forced expiratory volume in one second (FEV_1_) to forced vital capacity of <0.70 or evidence of emphysema on chest computed tomography. Exclusion criteria were inability to ambulate and occurrence of an AE within 4 weeks of enrollment [15].

At baseline, information about demographics, medical history, and medications was obtained. Subjects reported being at their usual clinical status at the time of enrollment. Participants underwent measurement of FEV_1_, using an Eaglet spirometer (nSpire Health, Inc.) [28]. The 6MWT was performed following ATS guidelines, except that a practice 6MWT was not done [29]. Patient-reported measures included assessments of dyspnea using the modified Medical Research Council (MMRC) scale [30], HRQL using the SGRQ, with scores ranging from 0–100 and lower scores indicating better HRQL [31], and depression using the Beck Depression Inventory [32]. The SGRQ has been recommended for use as a patient-reported measure of functional status, and we present the SGRQ-AS [3]. We considered a history of previous AEs as a potential confounder. To assess the past occurrence of AEs, participants were asked if they had received treatment with prednisone for breathing problems in the year prior to enrollment [13], [15], [23], [33].

The StepWatch Activity Monitor (SAM) (Orthocare Innovations, Seattle, WA, USA), an ankle-worn accelerometer, measures step counts from all walking–as part of PA and exercise in persons with COPD. We have shown the SAM to be accurate and valid in persons with COPD [27]. In this study, participants wore the SAM for 14 consecutive days and were instructed to perform their *usual* physical activities and exercise. The SAM does not provide on-instrument feedback of steps walked. Subjects returned the SAM to study staff by mail. Study staff downloaded the step counts, which are date and time stamped, via a docking station. No-wear days, defined as ones with <200 steps recorded and <8 hours of wear time, were excluded from the analysis [27], [34]. Subjects with ≥8 no-wear days were excluded.

### Study Outcomes

The primary outcomes were AEs and COPD-related hospitalizations. AE was defined as “a complex of respiratory symptoms (increased or new onset) of at least two of the following: cough, sputum, wheezing, dyspnea, or chest tightness lasting 3 or more days, requiring a course of treatment with antibiotics or systemic steroids [15], [35].” We included moderate AEs that required treatment in the outpatient setting and severe AEs that required hospitalizations. We included hospitalizations due to AE and/or pneumonia as COPD-related hospitalizations. Hospitalizations due to other pulmonary or cardiac causes were excluded. After the baseline visit, participants were prospectively queried on the use of oral corticosteroids and/or antibiotics and hospitalizations for lung problems using a structured telephone interview every 3 months for a median of 16 months. Participant reports were verified with medical records. Two investigators, blinded to baseline characteristics, reviewed subject responses and medical records to determine if an AE had occurred and the primary cause of hospitalizations. Independence of events was assured by considering a new event only if subjects had been off oral corticosteroids and/or antibiotics for at least 14 days following the previous AE or COPD-related hospitalization [15], [36].

### Statistical Analysis

Descriptive results are reported as means ± SD or percentages, as appropriate. Comparisons of descriptive characteristics were performed with the use of unpaired T tests or Fisher's Exact Test, as appropriate. The incidence rates of AEs and COPD-related hospitalizations were determined by dividing the numbers of AEs and COPD-related hospitalizations by the person-years of follow-up. Predictors of AEs and COPD-related hospitalizations were assessed using negative binomial models with the logarithm of observation time as an offset variable (PROC GENMOD, SAS 9.2, SAS Institute; Cary, NC) [23], [36]. In this approach, daily step count was examined as a continuous variable and the rate ratio calculated by exponentiating the regression coefficient. In separate models, daily step count was categorized in quartiles. Linear trend *P* values were derived using an ordinal variable coded based on the median of each step-count quartile. Variables significant at the *P*<0.05 level in univariate analyses were subsequently examined in multivariate models. All multivariate models included FEV_1_ % predicted and prednisone for AE in previous year as covariates. To put the results into clinical context, we calculated the rate ratios and 95% confidence intervals corresponding to published minimum clinically important differences (MCIDs) for the 6MWT and SGRQ-AS [4], [37]–[40].

## Results

### Study Participants

A total of 188 persons with COPD were enrolled. The analysis excluded 12 subjects who did not have baseline step-count data because 5 were noncompliant with step-count monitoring (had ≥8 no-wear days), 5 had no baseline step-count data due to an AE during the monitoring period, 1 lost the SAM, and 1 had SAM accuracy <90%. An additional 7 subjects did not participate in follow-up telephone calls. There were no differences in FEV_1_ % predicted, 6MWT distance, SGRQ Total Score (SGRQ-TS), SGRQ-AS, or MMRC dyspnea score among the 19 subjects excluded and the 169 subjects included in the analysis. In 169 persons with baseline and follow-up data, 167 were males, mean age was 71±8 years, mean FEV_1_ was 1.55±0.57 L (54±20% predicted) [41], and mean daily step count was 5804±3141 (Table 1). All 4 GOLD stages were represented; most subjects were GOLD II (46%) and GOLD III (33%) [2]. Twenty subjects (12%) had participated in a previous pulmonary rehabilitation program and 43 (25%) used supplemental oxygen. Four subjects died during follow-up.

**Table 1:** Subject Characteristics.*

	Total	Mean Daily Step<5232+	Mean Daily Step≥5232+
	n = 169	n = 85	n = 84
Age†	71±8	73±8	69±8
Body-mass index†	29±6	30±7	28±5
Marital status†			
Married	76 (45)	46 (54)	30 (36)
Not married	93 (55)	39 (46)	54 (64)
Race			
White	156 (92)	79 (93)	77 (92)
Non-White	13 (8)	6 (7)	7 (8)
Employment status†			
Full or part-time	20 (12)	5 (6)	15 (18)
Not working	42 (25)	18 (21)	24 (28)
Retired	107 (63)	62 (73)	45 (54)
Education			
Some/Completed high school	76 (45)	40 (47)	36 (43)
Some/Completed college or higher	93 (55)	45 (53)	48 (57)
Alcohol use			
≥1 day/week	52 (31)	30 (35)	22 (26)
<1 day/week	117 (69)	55 (65)	62 (74)
Prior participation in pulmonary rehabilitation†	20 (12)	16 (19)	4 (5)
Supplemental oxygen use†	43 (25)	32 (38)	11 (13)
Prednisone for AE in previous year	51 (30)	31 (36)	20 (24)
Coronary artery disease†	63 (37)	39 (46)	24 (29)
Congestive heart failure	23 (14)	15 (18)	8 (10)
Diabetes mellitus	48 (28)	24 (28)	24 (29)
Pack-years	68±37	70±33	66±40
FEV_1_ (liters)†	1.55±0.57§	1.42±0.57	1.68±0.55¶
FEV_1_, % predicted†	54±20§	51±20	58±20¶
GOLD stage†			
I	16 (10)§	6 (7)	10 (12)¶
II	77 (46)	35 (41)	42 (51)
III	56 (33)	28 (33)	28 (34)
IV	19 (11)	16 (19)	3 (4)
6MWT distance (meters)†	371±100	319±90	423±81
MMRC dyspnea score†			
0–1	68 (40)	20 (24)	48 (57)
2–4	101 (60)	65 (76)	36 (43)
SGRQ-TS†	45±20	49±18	42±20
SGRQ-AS†	63±23	69±19	57±25
Beck depression index	12±11	11±11	12±11
Medication for COPD‡			
Any short-acting β_2_ agonist	150 (89)	78 (92)	72 (86)
Any short-acting muscarinic antagonist	29 (17)	18 (21)	11 (13)
Any long-acting β_2_ agonist	108 (64)	56 (66)	52 (62)
Any long-acting muscarinic antagonist	125 (74)	63 (74)	62 (74)
Any inhaled corticosteroid	115 (68)	61 (72)	54 (64)

AE denotes acute exacerbation; FEV_1_ forced expiratory volume in 1 second; GOLD Global Initiative for Chronic Obstructive Lung Disease; 6MWT 6-minute walk test; MMRC Modified Medical Research Council; SGRQ-TS St. George's Respiratory Questionnaire Total Score; and SGRQ-AS St. George's Respiratory Questionnaire Activity Score.

* Mean ± standard deviation for continuous variables and N (%) for categorical variables.

+ The median average daily step count is 5,232.

† *P* Value<0.05; Unpaired T-test (continuous variables) or Fisher's Exact Test (categorical variables).

‡ Information on medication was self-reported; subjects may have been taking more than one medication.

§ N = 168.

¶ N = 83.

**Figure 1:**
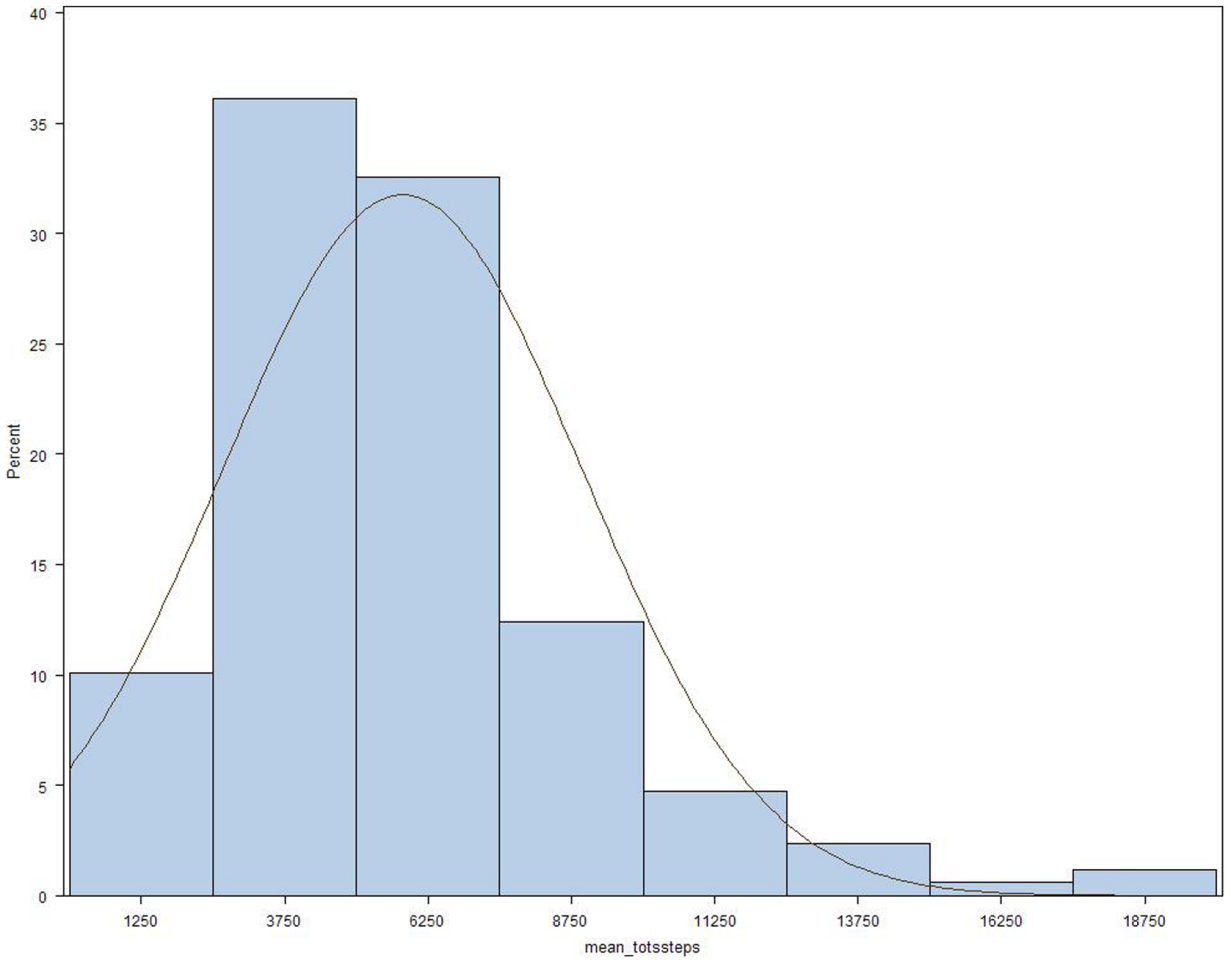
Distribution of mean daily step count. Median is 5,232 steps per day, N = 169.

Figure 1 shows the distribution of average daily step count. Of 2,366 days monitored, only 3% (n = 81) met the definition of a no-wear day. Compared to subjects with average daily step count ≥ the median average of 5,232, those with a daily step count <5,232 were significantly older, and had higher body-mass index (BMI), lower FEV_1_ % predicted, lower 6MWT distance, higher MMRC dyspnea score, worse SGRQ-TS, worse SGRQ-AS, and higher frequency of supplemental oxygen use and coronary artery disease (Table 1). In the 51 subjects (30%) who had used prednisone for breathing problems in the year prior to enrollment, mean daily step count was 4956±2608, which was significantly lower than the 6170±3288 steps per day observed in the 118 subjects who had not used prednisone (*P* = 0.012).

### Outcome Assessment

Over 209 person-years of follow-up, there were 263 AEs (incidence rate 1.3±1.6 per person-year) in 99 of 169 subjects (59%). Of these, 167 AEs were experienced by 54 of the 85 persons with daily step count < the median, and 96 AEs were experienced by 45 of the 84 persons with daily step count ≥ the median. There were 116 COPD-related hospitalizations (incidence rate 0.56±1.09 per person-year) in 54 of 169 subjects (32%). Of these, 79 hospitalizations were experienced by 37 persons with daily step count < the median, and 37 hospitalizations were experienced by 17 persons with daily step count ≥ the median. 224 AEs (85%) and 108 COPD-related hospitalizations (93%) were verified with medical records.

In univariate models, lower daily step count was a significant predictor of higher rates of future AEs and COPD-related hospitalizations (Table 2). There was a significant linear trend of decreasing daily step count by quartiles and increasing rate ratios for AEs (*P* = 0.0003) and COPD-related hospitalizations (*P* = 0.0003). Lower 6MWT distance and worse SGRQ-AS were also significantly associated with higher rate ratios for AEs and COPD-related hospitalizations (Table 2). Lower FEV_1_ % predicted and prednisone for AE in previous year were significantly associated with a higher rate ratio for AEs and COPD-related hospitalizations. Worse SGRQ-TS and supplemental oxygen use also significantly predicted future AEs. Age, BMI, pack-years, history of diabetes mellitus or coronary artery disease, MMRC dyspnea score, Beck's depression index, and season were not related to the risk of AEs and COPD-related hospitalizations.

**Table 2:** Univariate Associations with Number of Acute Exacerbations and COPD-Related Hospitalizations.

Characteristics	Acute Exacerbations			COPD-Related Hospitalizations		
	Rate Ratio	95% CI	*P* Value	Rate Ratio	95% CI	*P* Value
Age (per year increase)	1.02	0.99–1.04	0.13	1.03	0.995–1.07	0.08
Body-mass index (per kg/m^2^ increase)	0.996	0.96–1.03	0.81	0.96	0.92–1.01	0.16
Mean Daily Step Count (per 1000 step decrease)	1.11	1.04–1.19	0.003	1.29	1.13–1.49	0.0003
Mean daily step Quartiles						
(ref ≥6956)						
<3667	3.00	1.68–5.36	0.0002	8.69	2.92–25.8	<0.0001
3667≤×<5232	2.62	1.46–4.71	0.001	6.94	2.31–20.9	0.0006
5232≤×<6956	2.36	1.30–4.27	0.005	6.80	2.25–20.6	0.0007
*P* for linear trend			0.0003			0.0003
6MWT distance	1.10	1.03–1.17	0.003	1.21	1.10–1.34	0.0002
(per 30-meter decrease§)						
SGRQ-AS (per 4-point worsening§)	1.07	1.03–1.12	0.0005	1.12	1.04–1.19	0.002
FEV_1_, % predicted†	1.13	1.02–1.25	0.01	1.22	1.05–1.42	0.008
(per 10% decrease in % of predicted value)						
Prednisone for AE in previous year (ref = no)	2.44	1.66–3.58	<0.0001	2.16	1.17–4.00	0.01
SGRQ-TS	1.07	1.03–1.12	0.002	1.09	1.01–1.17	0.02
(per 4-point worsening)						
MMRC dyspnea score 2–4 (ref = 0–1)	1.47	0.97–2.22	0.07	1.67	0.89–3.14	0.11
Supplemental oxygen use (ref = no)	1.56	1.01–2.40	0.04	1.50	0.77–2.91	0.23
Pack-years	1.003	0.998–1.01	0.27	1.001	0.99–1.01	0.87
Diabetes mellitus						
(ref = no)	1.14	0.73–1.79	0.55	1.23	0.63–2.41	0.54
Coronary artery disease	1.08	0.71–1.65	0.72	0.90	0.47–1.73	0.75
(ref = no)						
Beck depression index	1.01	0.99–1.02	0.50	1.005	0.98–1.03	0.74
Season of step count monitoring						
(ref = Summer)						
Fall	0.85	0.51–1.40	0.52	0.98	0.45–2.13	0.97
Winter	0.80	0.40–1.61	0.54	0.61	0.20–1.88	0.39
Spring	0.79	0.44–1.41	0.43	1.14	0.48–2.73	0.76

6MWT denotes 6-minute walk test; SGRQ-AS St. George's Respiratory Questionnaire Activity Score; FEV_1_ forced expiratory volume in 1 second; AE acute exacerbation; SGRQ-TS St. George's Respiratory Questionnaire Total Score; MMRC Modified Medical Research Council; and ref reference group.

§ Rate ratios calculated for a MCID of 30 m [37] for 6MWT and 4 units [42] for SGRQ-AS. The regression coefficients (SE) in natural log risk per 30-m decrease in 6MWT predicting AEs and COPD-related hospitalizations are 0.0976 (0.0323) and 0.1946 (0.0516), respectively. The regression coefficients (SE) in natural log risk per 4-unit decrease in SGRQ-AS predicting AEs and COPD-related hospitalizations are 0.0712 (0.0206) and 0.1091 (0.0347), respectively.

† N = 168.

In multivariate models adjusting for FEV_1_ % predicted and prednisone for AE in previous year, for each 1000 fewer steps per day walked at baseline, there was a significantly increased rate of AEs (rate ratio 1.07; 95%CI = 1.003–1.15) and COPD-related hospitalizations (rate ratio 1.24; 95%CI = 1.08–1.42) (Table 3). The rate ratio for AEs and COPD-related hospitalizations in each step-count quartile was significantly increased compared to the rate ratio for persons in the highest step-count quartile (Table 3). Compared to persons in the highest step-count quartile, persons in the lowest quartile had a rate ratio of 2.26 (95%CI = 1.25–4.08) for AEs and 6.01 (95%CI = 1.99–18.2) for COPD-related hospitalizations. There was a significant linear trend of decreasing daily step count by quartiles and increasing rate ratios for AEs (*P* = 0.008) and COPD-related hospitalizations (*P* = 0.003).

**Table 3:** Multivariate Models of Associations between Daily Step Count and Number of Acute Exacerbations and COPD-Related Hospitalizations Adjusting for FEV_1_ % Predicted and Prednisone for AE in Previous Year.*

Model 1†	Acute Exacerbations				COPD-Related Hospitalizations			
	Rate Ratio	95% CI		*P* value	Rate Ratio	95% CI		*P* value
FEV_1_, % predicted (per 10% increase in % of predicted value)	1.05	0.95	1.16	0.33	1.14	0.98	1.32	0.09
Prednisone for AE in previous year (ref = no)	2.17	1.48	3.18	<0.0001	1.72	0.94	3.13	0.08
Mean Daily Step Count (per 1000 step decrease)	1.07	1.003	1.15	0.04	1.24	1.08	1.42	0.003

Model 2†	Acute Exacerbations				COPD-Related Hospitalizations			
	Rate Ratio	95% CI		*P* value	Rate Ratio	95% CI		*P* value
FEV_1_, % predicted (per 10% increase in % of predicted value)	1.04	0.94	1.15	0.41	1.15	0.99	1.34	0.07
Prednisone for AE in previous year (ref = no)	2.12	1.45	3.1	0.0001	1.65	0.92	2.99	0.1
Mean Daily Step Count in Quartiles								
(ref ≥6956)								
<3667	2.26	1.25	4.08	0.007	6.01	1.99	18.2	0.002
3667≤×<5232	2.11	1.19	3.74	0.01	6.04	2	18.19	0.001
5232≤×<6956	1.96	1.09	3.51	0.02	5.04	1.65	15.41	0.004
*P* for linear trend				0.008				0.003

FEV_1_ denotes forced expiratory volume in 1 second; AE acute exacerbation; and ref reference group.

* N = 168.

† Two separate multivariate models. Model 1 examines daily step count as a continuous variable. Model 2 examines daily step count in quartiles.

Similarly, in multivariate models, lower 6MWT distance and worse SGRQ-AS were significant predictors of AEs and COPD-related hospitalizations, independent of FEV_1_ % predicted and prednisone for AE in previous year (Tables 4 and 5). A decrease of 30 meters (37) in 6MWT distance was associated with an increased rate ratio of 1.07 (95%CI = 1.01–1.14) for AEs and 1.18 (95%CI = 1.07–1.30) for COPD-related hospitalizations. A worsening of SGRQ-AS by 4 points [42] was associated with an increased rate ratio of 1.05 (95%CI = 1.01–1.09) for AEs and 1.10 (95%CI = 1.02–1.17) for COPD-related hospitalizations. SGRQ-TS and supplemental oxygen use were not significantly associated with risk of AEs and COPD-related hospitalizations in multivariate models, adjusting for FEV_1_ % predicted and prednisone for AE in previous year.

**Table 4:** Multivariate Model of Associations between 6MWT distance and Number of Acute Exacerbations and COPD-Related Hospitalizations Adjusting for FEV_1_ % Predicted and Prednisone for AE in Previous Year.*

	Acute Exacerbations				COPD-Related Hospitalizations			
	Rate Ratio	95% CI		*P* value	Rate Ratio	95% CI		*P* value
FEV_1_, % predicted (per 10% increase in % of predicted value)	1.06	0.96	1.17	0.23	1.15	0.99	1.33	0.06
Prednisone for AE in previous year (ref = no)	2.14	1.46	3.14	0.0001	1.71	0.95	3.07	0.08
6MWT distance (per 30-meter decrease§)	1.07	1.01	1.14	0.03	1.18	1.07	1.30	0.001

FEV_1_ denotes forced expiratory volume in 1 second; AE acute exacerbation; ref reference group; and 6MWT denotes 6-minute walk test.

* N = 168.

§ Rate ratios calculated for a MCID of 30 m for 6MWT. The regression coefficients (SE) in natural log risk per 30-m decrease in 6MWT distance predicting AEs and COPD-related hospitalizations are 0.0674 (0.0308) and 0.1624 (0.0502), respectively.

**Table 5:** Multivariate Model of Associations between SGRQ-AS and Number of Acute Exacerbations and COPD-Related Hospitalizations Adjusting for FEV_1_ % Predicted and Prednisone for AE in Previous Year.*

	Acute Exacerbations				COPD-Related Hospitalizations			
	Rate Ratio	95% CI		*P* value	Rate Ratio	95% CI		*P* value
FEV_1_, % predicted (per 10% increase in % of predicted value)	1.07	0.97	1.17	0.17	1.17	1.02	1.36	0.03
Prednisone for AE in previous year (ref = no)	1.99	1.34	2.95	0.0006	1.60	0.87	2.94	0.13
SGRQ-AS (per 4-point worsening§)	1.05	1.01	1.09	0.02	1.10	1.02	1.17	0.008

FEV_1_ denotes forced expiratory volume in 1 second; AE acute exacerbation; ref reference group; and SGRQ-AS St. George's Respiratory Questionnaire Activity Score.

* N = 168.

§ Rate ratios calculated for a MCID of 4 units for SGRQ-AS. The regression coefficients (SE) in natural log risk per 4-unit decrease in SGRQ-AS predicting AEs and COPD-related hospitalizations are 0.0484 (0.0202) and 0.0923 (0.0347), respectively.

## Discussion

Our results demonstrate that persons with COPD with lower daily step count have significantly higher rate ratios for AEs and COPD-related hospitalizations, independent of FEV_1_ % predicted and previous exacerbation history. These novel findings are further supported by the significant linear associations over the entire range of daily step counts with rate ratios for AEs and COPD-related hospitalizations. Our results strongly support the rationale to study PA promotion as part of future exacerbation-prevention interventions in COPD.

A strength of our study is the use of 3 complementary measures of functional status to assess exacerbation risk prospectively in the same cohort. We examined daily step count as a direct measure of PA in the community, 6MWT as a clinic-based test of exercise capacity, and the SGRQ-AS as a patient-reported assessment of PA. Our data demonstrate that the relationship between PA and exacerbation risk is robust since daily step count predicts AE and COPD-related hospitalizations in a similar fashion as 6MWT distance and SGRQ-AS. Our results add to the evidence that daily step count is an important clinical characteristic of persons with COPD that can complement the 6MWT and questionnaire assessment of PA [43].

We focus on daily step count because it can be easily and directly translatable from the research to the clinical setting. Daily step count is a meaningful and relevant metric that, from the public health standpoint, can help define PA recommendations and promote PA in persons with COPD [44]. Healthcare providers and patients understand what it means to target PA goals to increase daily step counts. It is not feasible for providers to advise patients to improve their 6MWT distance since it is a clinic-based measure of exercise capacity that has little meaning to an individual. We previously published a pilot study showing that it is feasible for patients to monitor daily step count with a pedometer, and that persons with COPD can increase their walking with step-count goals [45].

In addition, directly measured daily step count overcomes limitations of questionnaire assessments of PA. First, it is well-known that persons overestimate self-reported physical activity. Second, the SGRQ-AS is used primarily in research settings and has no obvious meaning to most clinicians and all patients. Finally, prior studies using self-reported PA to examine risk for AEs/hospitalizations crudely characterized PA as ≥2 hours per week versus <2 hours per week [8], [25]. In contrast, directly measured daily step count allows accurate characterization of the relationship between PA and risk of AEs and hospitalizations so that future intervention studies can be appropriately designed.

To date, history of previous AEs has emerged as the strongest predictor of future AEs and hospitalizations, and FEV_1_ % predicted has been consistently found to be a significant predictor of future AEs and hospitalizations [13], [19]–[23]. However, spirometry alone is an inadequate predictor as there is a subset of patients with severely reduced FEV_1_ % predicted who do not experience frequent AEs [13]. We have identified daily step count, 6MWT distance, and SGRQ-AS as significant predictors of risk of AEs and COPD-related hospitalizations, independent of FEV_1_ % predicted. Furthermore, we show that lower daily step count, lower 6MWT distance, and worse SGRQ-AS predict future AEs and COPD-related hospitalizations, regardless of previous AE history. Our results that 6MWT and SGRQ-AS are independent predictors of risk of AEs and COPD-related hospitalizations are consistent with previously published studies. Two previous studies have shown that 6MWT distance predicts AEs in univariate, but not multivariate models [13], [46]. A lower 6MWT distance has been shown to predict hospitalizations in univariate but not multivariate models, which did not account for previous AEs or hospitalizations [19]. Epidemiological studies have shown a significant trend of decreasing physical activity, assessed by self-report, and increased risk of COPD-related admissions and hospitalization readmissions for COPD exacerbation [8], [9]. We calculated the rate ratios corresponding to a range of potentially clinically relevant changes in 6MWT to put our results into greater clinical context (Tables 4 and 6) [4], [37]–[40]. Similarly, we calculated the rate ratios that correspond to a 4-point worsening in SGRQ-AS. As MCID data for the SGRQ-AS are not available, we extrapolated the MCID of 4 units based on the SGRQ-TS [31], [42].

**Table 6:** Calculated Rate Ratios for Published MCIDs for 6MWT Distance.

6MWT Distance MCIDs	Acute Exacerbations		COPD-Related Hospitalizations	
	Rate Ratio	95% CI	Rate Ratio	95% CI
Per 25 meter decrease [4], [38]	1.058	1.006–1.112	1.145	1.055–1.243
Per 35 meter decrease [39]	1.082	1.008–1.161	1.209	1.078–1.356
Per 54 meter decrease [40]	1.129	1.013–1.258	1.340	1.122–1.599

MCID denotes minimum clinically important difference; and 6MWT 6-minute walk test.

A host of variables including chronic hypercapnia, pulmonary hypertension, hypoxemia, current smoking, older age, lower BMI, higher MMRC dyspnea score, and season have been inconsistently associated with AEs or hospitalizations in previous studies [19]–[23], [47]. We did not find an association between pack-years, age, BMI, MMRC dyspnea score, oxygen use, prior pulmonary rehabilitation, or season with AEs and COPD-related hospitalizations. Our results may reflect differences in age, gender, and FEV_1_ among cohorts and we accounted for potential confounding factors. Our study extends the generalizability and significance of the current literature by (1) including persons with all COPD severities, (2) studying moderate AEs as well as severe AEs, (3) directly measuring PA, and (4) including persons with COPD who have never participated in pulmonary rehabilitation [25], [26], [46].

Compared to other studies, our higher mean % predicted FEV_1_ is due to the fact that we included persons with all stages of COPD, including GOLD I or mild COPD. Our study was designed to be as inclusive as possible to increase generalizability, and thus included persons with all GOLD stages of COPD severity. Our cohort has similar frequencies of moderate COPD (GOLD II) and very severe (GOLD IV) as previously published clinical trials in COPD such as Evaluation of COPD Longitudinally to Identify Predictive Surrogate Endpoints (ECLIPSE) [13] and Understanding the Potential Long-term Impacts on Function with Tiotropium (UPLIFT) [48], supporting that our cohort represents the entire range of disease severity. Interestingly, our results are obtained in a cohort that has been largely medically optimized, indicating the importance of factors other than pharmacological therapy in managing exacerbation risk. Sixty-four percent of subjects were using a long-acting β_2_ agonist and 74% a long-acting muscarinic antagonist.

The main strengths of our study include our use of 3 complementary measures of functional status, our validated method of measuring daily step count, our structured approach to obtaining a prospective history of AEs and COPD-related hospitalizations, and the high percentage of events confirmed with medical records. We used an *a priori* event-based definition of AE that was easy for patients to recall and had blinded adjudication of events [15], [36]. Given our clear definition of AEs, every 3 month follow-up, and medical chart review, it is unlikely that we missed any AEs as we defined them. Much of the current literature has reported on risk factors only for severe AEs that resulted in hospitalizations [8], [9], [19], [20], [23], [25], [26]. Our inclusion of moderate AEs that required medication treatment but not hospitalization broadens the significance of our results, as most AEs do not result in hospitalizations. It is possible that we did not capture mild AEs not requiring treatment. Nevertheless, the incidence rate for AEs of 1.3 per person-year observed in our study is comparable to incidence rates reported in the placebo groups of large COPD clinical trials [13], [33], [48].

Some limitations need to be considered. We did not capture upper extremity activities. However, total daily PA has been shown to be closely related to leg activity in persons with COPD [49]. We did not measure the intensity of walking or activities such as swimming or bicycling that do not result in step counts. Previously, devices used to assess PA have reported relatively obscure units such as “activity units” which are difficult to understand and do not allow comparison between studies. The devices used in previous studies include (1) the RT3 accelerometer that reports physical activity movements using “vector magnitude units” (VMUs); (2) the Dynaport activity monitor that reports time spent in walking, cycling, standing, sitting, or lying; and (3) the SenseWear armband that reports total daily energy expenditure which is then converted to a “physical activity level” [6], [7]. Depew et al. have shown that daily step count is a surrogate for physical activity level, with daily step count <4,580 reflecting severe inactivity or physical activity level <1.40 [50]. Since walking is a common PA and steps per day is a measure of PA that is easy to understand, steps per day is a clinically relevant and practical variable to monitor in persons with COPD.

We did not track daily step count during follow-up, but we have previously shown that daily step count does not change significantly over time in stable COPD [27]. Therefore misclassification of baseline step counts is unlikely. The follow-up period, a median of 16 months, could be considered short, but it was sufficient to see a significant association between daily step count and risk of AEs and COPD-related hospitalizations. In addition, new events, such as a stroke or hip replacement, which may affect walking are more likely to occur with a longer follow-up period, confounding the relationship between daily step count and risk of AEs and COPD-related hospitalizations.

We considered a history of previous AEs requiring therapy with corticosteroids as a potential confounder since AEs tend to recur in the same person. To assess AEs in the year prior to study enrollment, we asked, ‘Have you used prednisone for breathing problems in the past year?’ This approach of adjustment for previous exacerbations has been used in previously published studies [13], [15], [23]. The report of prednisone use before study entry is distinct from the definition of AEs assessed prospectively, “a complex of respiratory symptoms (increased or new onset) of at least two of the following: cough, sputum, wheezing, dyspnea, or chest tightness lasting 3 or more days, requiring a course of treatment with antibiotics or systemic steroids,” as previously used in large clinical studies of COPD exacerbations [13], [15], [23].

We did not collect information on the time period between participation in a pulmonary rehabilitation program and participation in this study. In case the results are biased because the 20 subjects who had ever participated in pulmonary rehabilitation had an increased daily step count, we performed a sensitivity analysis excluding the 20 subjects. We found similar results, and thus, included the 20 subjects in the final results. Finally, these results need to be confirmed in larger studies, and intervention studies are needed to assess whether increases in daily step count reduce AE and COPD-related hospitalization risk in persons with COPD.

In conclusion, these results provide evidence for the importance of daily step count as a determinant of health status and exacerbation risk in persons with COPD. Our results suggest that there is a subgroup of COPD patients with low daily step count who have significantly increased risk of AEs and COPD-related hospitalizations, regardless of their % predicted FEV_1_. We speculate that the “low walker” may be a novel COPD phenotype. In contrast to other proposed phenotypes defined by frequent exacerbations, radiologic differences, or persistent systemic inflammation, the PA phenotype is potentially amenable to behavioral modification [13], [47], [51].
